# The spotted parrotfish genome provides insights into the evolution of a coral reef dietary specialist (Teleostei: Labridae: Scarini: *Cetoscarus ocellatus*)

**DOI:** 10.1002/ece3.11148

**Published:** 2024-03-12

**Authors:** Yi‐Kai Tea, Yulu Zhou, Kyle M. Ewart, Guo Cheng, Kazuhiko Kawasaki, Joseph D. DiBattista, Simon Y. W. Ho, Nathan Lo, Shaohua Fan

**Affiliations:** ^1^ School of Life and Environmental Sciences University of Sydney Sydney New South Wales Australia; ^2^ Australian Museum Research Institute Australian Museum Sydney New South Wales Australia; ^3^ State Key Laboratory of Genetic Engineering, Lab for Evolutionary Synthesis, Human Phenome Institute, Zhangjiang Fudan International Innovation Center, School of Life Science Fudan University Shanghai China; ^4^ Department of Anthropology Pennsylvania State University University Park Pennsylvania USA; ^5^ School of Environment and Science Griffith University Southport Queensland Australia

**Keywords:** adaptation to dietary toxins, coral reef fish, Labridae, Scarini, whole‐genome sequencing

## Abstract

With over 600 valid species, the wrasses (family Labridae) are among the largest and most successful families of the marine teleosts. They feature prominently on coral reefs where they are known not only for their impressive diversity in colouration and form but also for their functional specialisation and ability to occupy a wide variety of trophic guilds. Among the wrasses, the parrotfishes (tribe Scarini) display some of the most dramatic examples of trophic specialisation. Using abrasion‐resistant biomineralized teeth, parrotfishes are able to mechanically extract protein‐rich micro‐photoautotrophs growing in and among reef carbonate material, a dietary niche that is inaccessible to most other teleost fishes. This ability to exploit an otherwise untapped trophic resource is thought to have played a role in the diversification and evolutionary success of the parrotfishes. In order to better understand the key evolutionary innovations leading to the success of these dietary specialists, we sequenced and analysed the genome of a representative species, the spotted parrotfish (*Cetoscarus ocellatus*). We find significant expansion, selection and duplications within several detoxification gene families and a novel poly‐glutamine expansion in the enamel protein ameloblastin, and we consider their evolutionary implications. Our genome provides a useful resource for comparative genomic studies investigating the evolutionary history of this highly specialised teleostean radiation.

## Graphical abstract

The genome of the spotted parrotfish provides insight into the evolution of a coral reef specialist. Expansion and selection of detoxifying genes suggest a potential role in the metabolism of harmful dietary targets.

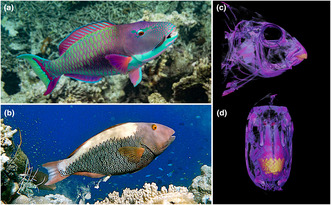

## 1 INTRODUCTION

The wrasses (family Labridae) have been described as one of the most stunning examples of trophic and morphological diversification in modern teleost fishes on coral reefs (Streelman et al., 2002; Westneat et al., 2005). With over 600 valid species, the family is the largest radiation in marine environments, second only to the Gobiidae (Fricke et al., 2023). The contemporary success of this major teleostean family can be attributed to a multitude of key morphological innovations in their oral and pharyngeal jaw apparatus, resulting in their ability to exploit nearly every trophic guild, including echinoderms, bivalves, gastropods, zooplankton, ectoparasites, coral mucus, detritus, and even algae (Evans et al., 2019; Huertas & Bellwood, 2020; Wainwright et al., 2004; Westneat et al., 2005). Consequently, considerable research attention has been directed towards the evolution of the Labridae in recent years.

Among labrid fishes, the parrotfishes (tribe Scarini) possess some of the most dramatic and specialized adaptations, unique with regards to their diet and associated ecomorphology. Unlike other labrids, dentition of the Scarini consists of abrasion‐resistant, biomineralized teeth fused into plates that are secondarily strengthened by a coating of enameloid (Marcus et al., 2017). This highly derived beak‐like oral dentition, coupled with a pharyngeal mill supported by powerful levator muscles, allows parrotfishes to reduce calcareous carbonate material rich in micro‐photoautotrophs on which they feed, particularly epilithic, endolithic, or endosymbiotic cyanobacteria (Bellwood & Choat, 1990; Clements et al., 2017; Nicholson & Clements, 2020, 2023a, 2023b; Wainwright et al., 2004).

The remarkable biology and morphology of the parrotfishes have attracted significant research attention (Price et al., 2011), including the development of genomic frameworks in which to explore their evolutionary histories and diversification rates (Kazancioglu et al., 2009). Despite the plethora of ecological studies centred around parrotfishes and their role in coral reefs (Bellwood et al., 2012; Lange et al., 2022; Perry et al., 2022), there has been disproportionately less scrutiny of their trophodynamics and precise feeding targets (Clements et al., 2017). Recent work integrating microhistology and DNA‐metabarcoding has not only quantified dietary targets of the parrotfishes but has also demonstrated that parrotfishes exhibit finer‐scale trophic partitioning between species (Nicholson & Clements, 2020, 2021, 2023a, 2023b). Given the degree of ecomorphological distinctiveness, their dominant presence on coral reefs, and the role that they play in reef bioerosion and sediment production (Boldando et al., 2014; Perry et al., 2022), parrotfishes serve as ideal candidates for exploring the complex interplay of evolutionary forces operating on coral reefs.

Recent advances in genomics aimed at addressing ecological and evolutionary questions have led to a rapid increase in the number of de novo genome assemblies of non‐model organisms (Kelley et al., 2016). Around 200 fish species have reference genome assemblies, the majority of which are teleosts (Rhie et al., 2021). Yet there is a surprising dearth of genomes from taxa inhabiting coral reefs, with only a handful of high‐quality genomes available from the family Labridae (Liu et al., 2021). In order to better understand the evolutionary drivers responsible for the success of this lineage, we sequenced and analysed the genome of the spotted parrotfish (*Cetoscarus ocellatus*), a representative species among the parrotfishes notable for having a diet comprising live coral and epilithic and endolithic cyanobacteria (Nicholson & Clements, 2023b). From an evolutionary perspective, a genomic resource from *C. ocellatus* can provide valuable insight into the mechanisms behind some of the remarkable biology displayed by the parrotfishes, since few genomes from the Scarini have been made available.

Our findings reveal significant expansion, selection and duplications within several gene families responsible for detoxification, including the cytochrome P450 gene family and other non‐cyp450 carboxylesterases, suggestive of the spotted parrotfish's ability to neutralize toxins potentially associated with cyanobacterial filaments and other dietary targets. We find preliminary evidence that the structural mechanism responsible for the extreme hardness and biomineralization of parrotfish teeth may be a result of a previously unreported poly‐glutamine expansion in the enamel protein ameloblastin. We also reveal expansion and selection in several genes related to pigmentation and sex change. Together, these results highlight the complex interplay of evolutionary diversification and sexual selection operating in coral reef ecosystems.

## 2 MATERIALS AND METHODS

### 2.1 DNA extraction and library preparation

We obtained 50 mg of flash‐frozen muscle tissues of the spotted parrotfish from the Australian Museum Research Institute's tissue collection (specimen voucher AMS I.49509‐002). Tissue samples were crushed using a sterile pestle in a 1.5 mL Eppendorf tube containing 600 μL SDS Buffer and 20 μL Proteinase K (Roche, Switzerland) and incubated at 56°C overnight. Following tissue lysis, 5 μL of RNAse A (20 mg/mL) was added and incubated at 37°C for 1 h. We extracted DNA using the phenol/chloroform method and checked the concentration using Qubit fluorometric quantitation (ThermoFisher, USA). DNA quality was visualized using 1% agarose gel electrophoresis.

Genomic libraries were prepared according to the manufacturer's instructions. The single‐molecule real‐time sequencing bell (SMRT Bell) library was prepared using a PacBio DNA Template Prep Kit 1.0 (Pacific Biosciences). DNA quantitation was performed using a 2100 Bioanalyzer (Agilent Technologies). The SMRT Bell‐polymerase complex was constructed using a PacBio Binding Kit 2.0 (Pacific Biosciences), following the manufacturer's instructions. The library was loaded onto SMRT cells (Sequel SMRT Cell 1M v2; Pacific Biosciences) and sequenced using Sequel Sequencing Kit 2.1.

Transposase Enzyme‐Linked Long‐read Sequencing (TELL‐seq™; Chen et al., 2020) data (i.e. long‐range linked‐read data) were generated by the Australian Genome Research Facility (AGRF). TELL‐seq libraries were constructed using a TELL‐seq WGS Library Prep Kit (Universal Sequencing Technology). DNA extracted from the spotted parrotfish was incubated with ~8 million TELL beads for barcoding in a 0.2 mL PCR tube according to the manufacturer's protocol (performed by AGRF). Each TELL bead comprises 50,000 copies of at least one barcode sequence conjugated to its surface. Following barcoding, an eight‐cycle amplification step was carried out in order to generate libraries for sequencing.

Hi‐C library preparation was performed using the Arima Hi‐C Plus kit (Arima, USA) following the manufacturer's protocol for large animal tissue. Approximately 50 mg of fresh muscle tissue was flashed frozen and pulverized using liquid nitrogen. A crosslinking buffer containing 1% formaldehyde was added to the pulverized tissue to crosslink the DNA. We used 500 ng of DNA for Hi‐C library preparation. The final library preparation for sequencing was performed using the KAPA Hyper Prep kit following the protocol detailed in the Arima Hi‐C Plus kit. The libraries were quantified using a Qubit fluorometer (ThermoFisher, USA) and Bioanalyzer (Agilent, USA).

In order to estimate the number of available Hi‐C reads in the library, we first sequenced the library on the Illumina Miseq platform (Illumina, USA) using a MiSeq V2 Nano flow cell with 2 × 150 bp specification. The sequencing data were then processed using qc3C (DeMaere & Darling, 2021). The qc3C software assessed the Hi‐C library quality by calculating proximity ligation events which create k‐mers that would not naturally occur in the sample. Based on the qc3C result, we estimated the sequencing data needed for the library. Sequencing was carried out on the NovaSeq platform (Illumina, USA) using NovaSeq S4 flow cell 2 × 150 bp at Novogene (USA).

### 2.2 Genome assembly

An initial de novo assembly of the PacBio long‐read data was performed using Canu v.2.1.1 (Koren et al., 2017) with default settings and an estimated genome size of 1.4 Gb (based on published labrid genomes; Liu et al., 2021; Mattingsdal et al., 2018). Putative regions of haplotypic duplication and heterozygous overlaps were identified and removed based on sequence similarity and read depth using purge_dups v1.2.5 (Guan et al., 2020). We used minimap2 v2.18 (Li, 2018) to align raw PacBio reads to the draft assembly. The assembled genome was then polished using the PacBio reads with the program gcpp (https://github.com/PacificBiosciences/gcpp), implementing the ‘arrow’ algorithm, with the minimum coverage cut‐off required for variant calling set to 20, the maximum coverage set to 80, and the minimum confidence for a variant call set to 20.

TELL‐seq linked reads were used to scaffold the draft de novo long‐read assembly and improve its contiguity. Raw TELL‐seq reads were demultiplexed and processed using Tell‐Read (Chen et al., 2020). To ensure that TELL‐seq reads were compatible with downstream programs that utilize 10X linked‐read data, barcodes were downsized to 4,000,000 barcodes, compressed, and renamed using ust10x (provided with the Tell‐Read software). Barcodes were further processed and trimmed and sequences interleaved with Long Ranger basic v2.2.2. For all Long Ranger runs, the barcode whitelist was replaced to match TELL‐seq barcodes. Putative scaffolding graphs were generated using ARCS v1.2 (Yeo et al., 2018) and joined using LINKS v1.8.7 (Warren et al., 2015) with parameters ‘arcs‐make arcs *c* = 3 *a* = 0.9 *l* = 3 *z* = 500 *e* = 70,000 *m* = 10–250’. The optimal settings were identified through a parameter sweep with ARKS/LINKS.

Hi‐C reads were aligned to the ARCS/LINKS‐scaffolded draft assembly with bwa mem v0.7.17 (Li & Durbin, 2009) using the ‘‐SP5M’ flags (Table S1). PCR duplicates were removed using samtools v1.6 (Danecek et al., 2021). The resulting alignment was converted to a ‘bed’ file using bedtools v2.29.2 (Quinlan & Hall, 2010). Contig lengths were calculated using samtools. Scaffolding was performed using the program SALSA v2.3 (Ghurye et al., 2019), with the restriction sites set as ‘GATC’ and ‘GANTC’. A Hi‐C contact map was generated by converting SALSA2 outputs with juicer tools pre v1.22.1 and visualized using Juicebox v2.04.06 (Figure S1; Durand et al., 2016).

Polishing with long‐ and linked‐read data was conducted on the Hi‐C scaffolded assembly. A second round of polishing using the long‐read PacBio data was performed as described above (i.e. implementing the ‘arrow’ algorithm). Two further rounds of polishing were conducted with the TELL‐seq linked reads. The reads were aligned to the draft assembly with Long Ranger align v2.2.2, then scaffolds were split and variants were called in each scaffold using FreeBayes v0.9.21 (Garrison & Marth, 2012) with default settings. Variants were concatenated, normalised and filtered to retain only homozygous ALT and non‐REF sites. Consensus sequences were generated with bcftools v1.9.

To evaluate the contiguity of the spotted parrotfish genome (i.e. assessing the genome and scaffold lengths), we analysed the assembled scaffolds with QUAST v4.3 (Gurevich et al., 2013). To evaluate the completeness of the genome, we implemented a Benchmarking Universal Single‐Copy Orthologues (BUSCO) v3.0 analysis with default parameters, based on a core set of genes of the Actinopterygii database.

### 2.3 Genome annotation and repeat analysis

Genome annotation was performed using Fgenesh++ v7.2.2 (Salamov & Solovyev, 2000; Solovyev et al., 2006), hosted on a virtual machine at the Pawsey Supercomputing Research Centre (Australia). A repeat database was first generated using RepeatModeler v1.0.8 (Flynn et al., 2020). Repeats were subsequently masked in the genome assembly using the program RepeatMasker v4.0.6 (Smit et al., 2015), implementing the ‘‐nolow’ option. The masked and unmasked genome assemblies were used as inputs for Fgenesh++. Annotation using Fgenesh++ was performed with optimized gene‐finding parameters trained on the genome of the three‐spined stickleback (*Gasterosteus aculeatus*) and with non‐mammalian general pipeline parameters. We utilized the NCBI animal protein database, curated by Softberry (Solovyev et al., 2006), for the ‘prot_map’ homology‐based predictions. For genomic regions that did not contain ‘prot_map’ predictions, ab initio predictions were performed. The ab initio predictions were then used in a BLAST search against the NCBI database, and predictions without any matches were excluded from subsequent analyses.

Homologous snRNAs and miRNAs were predicted by searching the Rfam database (Kalvari et al., 2018) using Infernal v1.1.2 (Nawrocki & Eddy, 2013) with settings cmscan ‐Z 2735.647178 ‐‐cut_ga ‐‐rfam ‐‐nohmmonly. tRNAs and rRNAs were predicted using tRNAscan‐SE v2.0.8 (Lowe & Eddy, 1997) and RNAmmer v1.2 (Lagesen et al., 2007), respectively, with default settings.

### 2.4 Orthology assignment and gene family analysis

The coding sequences of 14 species, including coelacanth (*Latimeria chalumnae*) (Amemiya et al., 2013), human (*Homo sapiens*) (Lander et al., 2001), spotted gar (*Lepisosteus oculatus*) (Braasch et al., 2016), zebrafish (*Danio rerio*) (Howe et al., 2013), Nile tilapia (*Oreochromis niloticus*) (Brawand et al., 2014), Japanese medaka (*Oryzias latipes*) (Kasahara et al., 2007), three‐spined stickleback (*G. aculeatus*) (Jones et al., 2012), Japanese pufferfish (*Takifugu rubripes*) (Aparicio et al., 2002), oceanic sunfish (*Mola mola*) (Pan et al., 2016), and gilthead seabream (*Sparus aurata*) (Pérez‐Sánchez et al., 2019) were downloaded from the Ensembl database v107 (Cunningham et al., 2022). We downloaded the coding sequences of the copperband butterflyfish (*Chelmon rostratus*) (Fan et al., 2020), New Zealand spotty (*Notolabrus celidotus*), ballan wrasse (*Labrus bergylta*) (Lie et al., 2018) and humphead wrasse (*Cheilinus undulatus*) (Liu et al., 2021) from the NCBI RefSeq database (O'Leary et al., 2016). The proteome and coding sequences of the corkwing wrasse (*Symphodus melops*) were downloaded from https://figshare.com/articles/dataset/maker_aed‐1_0_pfam_eggnog_gff/5589997 and https://figshare.com/articles/dataset/Symphodus_melops_fasta/5590003.

We extracted the longest isoform per gene using get_longest_transcript.py. We inspected the spotted parrotfish proteome and ensured that there were no alternative isoforms. Orthologous proteins among the 16 species were identified using OrthoFinder v2.5.4 using ‐S diamond ‐M msa ‐T raxml ‐t 8 ‐a 8 (Emms & Kelly, 2019). Orthogroups were annotated according to the longest protein sequence in each orthogroup by eggNOG‐mapper with default settings.

We tested for expansions of gene families under a birth–death model using CAFE v5.0 (Mendes et al., 2020), using a dated species tree (see below) and gene counts from OrthoFinder as inputs. Highly variable gene families were excluded to avoid errors in parameter predictions. We used cafetutorial_clade_and_size_filter.py to filter out gene families that contained any species with over 100 copies of that gene. A time‐calibrated tree was constructed with r8s (http://loco.biosci.arizona.edu/r8s/) using the SpeciesTree_rooted.txt generated by OrthoFinder as input. Node calibrations used were divergence times between two species from Timetree (http://timetree.org/home).

### 2.5 Inference of genes that have undergone positive selection

To prepare and align sequences from the 16 target species for positive selection analysis, we first used MACSE v1.2 (Ranwez et al., 2011) to remove stop codons and correct for potential frameshift mutations in the coding sequences that were potentially introduced during alignment trimming. The coding sequences were aligned using PRANK v.170427 (Löytynoja & Goldman, 2010) using codon‐aware mode. We removed the poorly aligned regions using BMGE v1.12 (Criscuolo & Garibaldo, 2010) with codon‐aware mode.

We used the optimized branch‐site model (Yang & Nielsen, 1998; Zhang et al., 2005) in CodeML (Yang, 2007) to detect signatures of positive selection in the protein‐coding genes of the spotted parrotfish. In the branch‐site model, the null hypothesis assumes that all branches or all branches and codons evolve neutrally (*ω* ≈ 0), while the alternative model allows a subset of sites in a specific lineage to have a different *ω* ratio or accelerated nonsynonymous substitution rate (Yang, 1998; Yang & Nielsen, 1998; Zhang et al., 2005). Based on the results of the branch‐site models, we calculated the *p* values based on likelihood ratio tests and corrected the *p* values using an FDR approach (https://github.com/StoreyLab/qvalue). Genes with an FDR‐adjusted *p*‐value of < .2 were identified as undergoing positive selection (Tong et al., 2021).

To explore the functional impact of the positively selected genes or expanded gene families in the spotted parrotfish genome, we ran a protein sequence annotation using eggNOG‐mapper (Cantalapiedra et al., 2021; Huerta‐Cepas et al., 2019) and conducted a gene‐based gene ontology (GO) or a Kyoto Encyclopedia of Genes and Genome Pathways (KEGG) signal pathway enrichment test using clusterProfiler v4.2.2 (Wu et al., 2021) in RStudio. We used genes from the zebrafish as a representation of each gene family.

### 2.6 Identification of *ambn* and other *scpp* genes

In order to investigate the evolution of the parrotfish's unique dentition, we investigated the evolution of key teeth and enamel forming genes in vertebrates. We manually identified *scpp* genes in the spotted parrotfish genome as well as the following teleost genomes: humphead wrasse, New Zealand spotty, gilthead seabream, Japanese pufferfish, and the three‐spined stickleback. We identified *scpp* genes using TBLASTN (Altschul et al., 1990) with default settings using amino acid sequences encoded by previously known *scpp* genes of teleosts and non‐teleost actinopterygians. Syntenic analysis was also used to identify *scpp* genes. Previous studies have shown that the arrangement of zebrafish *scpp* genes can be traced back to four *scpp* gene clusters that arose from two primordial clusters through teleost genome duplication (Braasch et al., 2016). In the present study, we first searched for genomic regions syntenic to these four *scpp* gene clusters using TBLASTN with amino acid sequences encoded by genes located adjacent to the four *scpp* gene clusters. For *scpp* genes from the humphead wrasse, New Zealand spotty, gilthead seabream, Japanese pufferfish, and three‐spined stickleback, we searched RNA‐seq intron‐spanning reads in these genomic regions, which are available in the gene database of GenBank. Using these *scpp* gene sequences, we subsequently identified *scpp* genes of spotted parrotfish with TBLASTN.

We investigated exon 4 of the ameloblastin gene (*ambn*) in the spotted parrotfish and five other labrid species: ballan wrasse (*Labrus bergylta*; GCF_900080235.1), cunner (*Tautogolabrus adspersus*; GCA_020745685.1), corkwing wrasse (*S. melops*; GCA_947650265.1), blue‐head wrasse (*Thalassoma bifasciatum*; GCA_008086565.1) and the sheepshead wrasse (*Semicossyphus pulcher*; GCA_022749685.1). These sequences were identified with TBLASTN in the whole‐genome shotgun contigs (wgs) database of GenBank.

Amino acid sequences encoded by *ambn* were manually aligned. Based on this amino acid sequence alignment, a gene tree was inferred using MEGAX by the maximum‐likelihood method (Kumar et al., 2018; Nei & Kumar, 2000). For phylogenetic inference, the best‐fit amino acid substitution model (JTT+I model) was also selected using MEGAX. All gaps were excluded from this analysis and support for internal branches was calculated by 500 bootstrap samples (Nei & Kumar, 2000).

### 2.7 Identification of pigmentation‐related genes

We collected a gene list associated with pigmentation. This list consists of 996 genes involved in pigmentation phenotypes that was complied with manual curation of genes annotated in HPO (https://hpo.jax.org/app/), Color Genes (http://www.ifpcs.org/colorgenes/) and genes reported in previous studies and combined with a cross‐species gene list of 650 genes for pigmentation biology in a review study (Baxter et al., 2018). In order to determine whether corresponding pigmentation‐related genes in fish were available, we used the BLAST function in NCBI database, resulting in the retention of 928 pigmentation‐related genes (PRG). Gene family expansions/contractions for these PRG based on the CAFÉ analyses were compared between the 16 analysed species.

### 2.8 Identification of detoxification‐related genes

We aligned the protein sequences of detoxification‐related genes using MAFFT v7.110 (Katoh & Standley, 2013) and trimmed the poorly aligned regions using trimAl v1.4.rev22 (Capella‐Gutiérrez et al., 2009) with default parameters. The substitution model of each gene family was inferred using prottest‐3.4.2 with default parameters. We inferred maximum‐likelihood gene trees with RAxML‐NG v1.0.3 (Kozlov et al., 2019) with 1000 bootstraps. The gene trees were visualized using iTOL (Letunic & Bork, 2021).

To detect syntenic relationships around the *ces2b* locus, collinear blocks among the 16 species in the present study were identified using MCScanX (Python version) (Tang et al., 2008). Specifically, we first obtained the longest isoform per gene sequence from the proteome of the 16 different species using ‘primary_transript.py’. We converted the GFF to a BED file using the jcvi.formats.gff script from MCScanX. The proteome sequences between two species were aligned using jcvi.compara.catalog with the ‘‐‐cscore = 0.99’ parameter. Syntenic genes around *ces2b* loci were then extracted and plotted with the jcvi.graphics.synteny script.

## 3 RESULTS

### 3.1 Genomic landscape and orthology assignment

Our assembly methods produced a 1.37 Gb genome assembled in 727 contigs, with an N50 of 4.98 Mb (Table S2). The assembly comprised 98.5% of the 3640 benchmarking universal single‐copy orthologues (BUSCOs) (Table S3), the highest value for any published teleost genome and comparable to that of the human assembly (Seppey et al., 2019). Our automated annotation pipeline annotated 33,835 genes (excluding ab initio annotations with no BLAST hits). Repetitive sequences account for 52% of the genome, with DNA transposons (Class II, 14.08% accounting for 192 Mb) more abundant than retroelements (Class I, 5.36% accounting for 73 Mb) (Figure 2a,b; Table S4). Among other available labrid genomes, this proportion of repetitive sequence is comparable only to the humphead wrasse (*C. undulatus*; DNA transposons 21.25%, accounting for 249 Mb; retroelements 3.82%, accounting for 45 Mb; Figure 2c). The proportion of DNA transposons and retroelements for two other labrid species, the ballan wrasse (*L. bergylta*) and corkwing wrasse (*S. melops*), were significantly lower than in the spotted parrotfish and humphead Maori wrasse (DNA transposons: 7.83% and 3.90%, accounting for 63 and 24 Mb; retroelements: 8.74% and 3.70% accounting for 70 and 23 Mb in ballan and corkwing wrasses, respectively; Figure 2d,e). When combined with the other 15 genomes, a total of 374,640 annotated genes (97%) were assigned to 21,832 orthogroups; 7641 orthogroups (35%) had all species present and 1882 were single‐copy orthogroups (8.62%; Figure S2).

We detected two waves of transposable element (TE) expansions (Figure 2b), characterized largely by DNA transposons, in the genome of the spotted parrotfish, and one wave of TE expansion in the humphead wrasse genome (Figure 2c). Our findings suggest that TE expansion resulting in genome expansion is likely to have occurred once in the common ancestor of the spotted parrotfish and humphead wrasse, but not within the labrini (no significant TE expansion was detected in the genomes of the ballan and corkwing wrasses).

### 3.2 Gene expansions and contractions

We identified 2190 and 502 gene families with signals of expansion or contraction, respectively, in the spotted parrotfish genome (Figure 3a), including significant expansion and significant enrichment (*p* < .05; Figure 3b) of eight gene families (*ces2*, *gsta*, *adh1*, *fmo5*, *cyp3a65*, *nat10l*, *cbr1*, and *gstm*; Figure S3) that were associated with xenobiotic metabolism. Our analyses also revealed duplications of *ces2* in the spotted parrotfish and Japanese pufferfish (*T. rubripes*) genomes, resulting in 12 copies of each, respectively (Figure 3c). These genes encode several carboxylesterases that are highly expressed in the liver and are responsible for the transformation and metabolism of xenobiotic compounds into more harmless metabolites (Tseng et al., 2005). Specifically, the duplicated copies of *ces2* in the spotted parrotfish and Japanese pufferfish genomes belong to the *ces2b* lineage and arose independently through species‐specific duplications (Figure 3c). Synteny analyses of the *ces2b* locus revealed that the neighbouring genes of *ces2b* are collinear in the genomes of the spotted parrotfish and the 12 other teleosts examined here, suggesting a conserved synteny at this locus during their evolutionary histories. These results also indicate that the extra copies of *ces2b* in the spotted parrotfish genome and the Japanese pufferfish genome likely arose through tandem duplication events (Figure 2d,e; Figures S4 and S5).

We find that ~25% (21 out of 83) of PRG families were significantly expanded in the spotted parrotfish genome, the highest among all teleost genomes compared in the present study (Figure 3f; Figure S6). We detected duplications of both *pax3a* and *pax3b* in the spotted parrotfish genome, resulting in four copies of *pax3* (versus two copies on average in other comparative teleosts; Figure S7), and a significant expansion of *chst8* in the spotted parrotfish (Figure 3e).

We identified 15 *scpp* genes in the spotted parrotfish, compared to between 12 and 20 *scpp* genes for the other teleost species (Figure 4a). The difference in the number of *scpp* genes in these species is mostly attributed to the number of *scpp3* genes. For example, two *scpp3* genes were identified in the spotted parrotfish, while eight *scpp3* genes were found in the gilthead bream (Figure 4a). Except for *scpp3* genes, the repertoire of *scpp* genes in the spotted parrotfish is similar to that of its phylogenetically closest relative, the humphead wrasse. We found no evidence of lineage‐specific *scpp* gene duplications in the spotted parrotfish or any of the other labrid species compared here, indicating that the suite of *scpp* genes is relatively conserved for the family.

### 3.3 Genes that have undergone positive selection

Of the 1882 single‐copy orthologues analysed using the branch‐site model, we inferred 143 (7.60%) genes that have undergone positive selection in the spotted parrotfish (Table S5). A GO enrichment analysis using clusterProfiler showed that the genes exhibiting signals of positive selection in the parrotfish genome are significantly enriched (FDR‐adjusted *p*‐value < .2) and are involved in biological processes associated with cellular structure organization (e.g. actomyosin structure organization, GO:0031032; extracellular matrix organization, GO:0030198; respiratory tube development, GO:0030323; and locomotion, GO:0040011). We also found that genes involved in focal adhesion, sex differentiation, and sex determination signalling pathways (KEGG: dre04510 *lamb2*, *lamc2*, *col9a2*, *kdr*, *pak7*, *igf1* and *rock1*) were significantly enriched (FDR‐adjusted *p*‐value < .2) based on the KEGG analysis.

## 4 DISCUSSION

### 4.1 A potential role in detoxification of dietary toxins

The diet of scarinin parrotfishes varies across species but largely includes epilithic, endolithic, epiphytic and endophytic micro‐photoautotrophs, particularly filamentous cyanobacteria such as *Calothrix* and *Lyngbya* morphotypes (Nicholson & Clements, 2023a). Unlike most other parrotfishes, the diet of the spotted parrotfish also includes sponges and live coral, the latter including hard coral from the genera *Pocillopora* (Nicholson & Clements, 2023b) and *Porites* (Bellwood & Choat, 1990; Bonaldo & Bellwood, 2011; Huertas et al., 2021). Recent analyses of substrata ingested by the spotted parrotfish based on core sampling and DNA barcoding suggest that the endosymbiotic micro‐photoautotrophs residing within live coral and sponges are likely to be the intended dietary targets, in which case spongivory and corallivory exhibited by the spotted parrotfish may be a dietary inconsequence (Nicholson & Clements, 2023b). Additionally, incidental dietary targets might include diatoms, fungi and dinoflagellates (Nicholson & Clements, 2023b).

Several of these dietary components are known to be chemically defended. *Pocillopora* and *Porites* have been hypothesized to produce allelochemicals and other biologically active metabolites that deter and impair predation from corallivorous specialists such as butterflyfishes (Gochfield, 2004). The toxicity of dietary micro‐photoautotrophs is less well understood, in part due to the dearth of biochemical studies and the polyphyletic nature of many cyanobacterial genera [e.g. *Lyngbya* (Berthold et al., 2022; Lefler et al., 2021)]. Nonetheless, several *Lyngbya* morphotypes are known to play a role in harmful cyanobacterial blooms (Albert et al., 2005) and are also known to deter grazing by fishes due to secondary metabolites (Capper et al., 2016; Matthew et al., 2010).

In most species of corallivorous fishes, detoxification of allelochemicals is aided through enzymes encoded by genes in the cytochrome P450 (*cyp*) superfamily (Maldonado et al., 2016; Parkinson & Ogilvie, 2010). These genes have been identified from various organisms across the tree of life and encode haeme‐thiolate enzymes that play central roles in detoxification through oxidative metabolism of a range of compounds including xenobiotics (Kirischian et al., 2011).

The expansions of several *cyp* and non‐*cyp*‐mediated genes in the spotted parrotfish genome are suggestive of a specialized diet as well as potentially playing a role in the detoxification and metabolism of harmful metabolites (Boušová et al., 2015; Contreras‐Zentella et al., 2022; Glisic et al., 2015; Liu et al., 2020; Tseng et al., 2005). This dietary specialization is also supported by the tandem duplication and maintenance of 12 copies of *ces2b* in the spotted parrotfish genome. These genes encode several carboxylesterases that are highly expressed in the liver and are responsible for the hydrolysis of carboxylesters into the corresponding alcohol and carboxylic acid (Hatfield et al., 2016; Satoh & Hosokawa, 1998, 2006; Tseng et al., 2005). Alternatively, given the wider variety of dietary targets of *Cetoscarus*, it is also possible that the spotted parrotfish exhibits a more generalist diet than other scarinin parrotfish species living on the same reef.

Duplication of *ces2* was also observed in the Japanese pufferfish, a species notorious for the bioaccumulation of tetrodotoxin (Noguchi & Arakawa, 2008). The diet of the Japanese pufferfish consists mostly of echinoderms, gastropods, crustaceans, cnidaria, as well as flatworms and ribbonworms, of which several are known to harbour tetrodotoxin, tetrodotoxin derivatives, saxitoxins and palytoxins (Miyazawa & Noguchi, 2001). It is possible that the increased production of *ces2* in the Japanese pufferfish plays a role in the detoxification or attenuation of these toxic metabolites. The expansion and duplication of several detoxification genes raises the question of whether the spotted parrotfish ingests toxins as part of its dietary repertoire, and therefore whether it is similarly able to detoxify these metabolites.

### 4.2 Pigmentation, sex change and their roles in sexual selection

**FIGURE 1:**
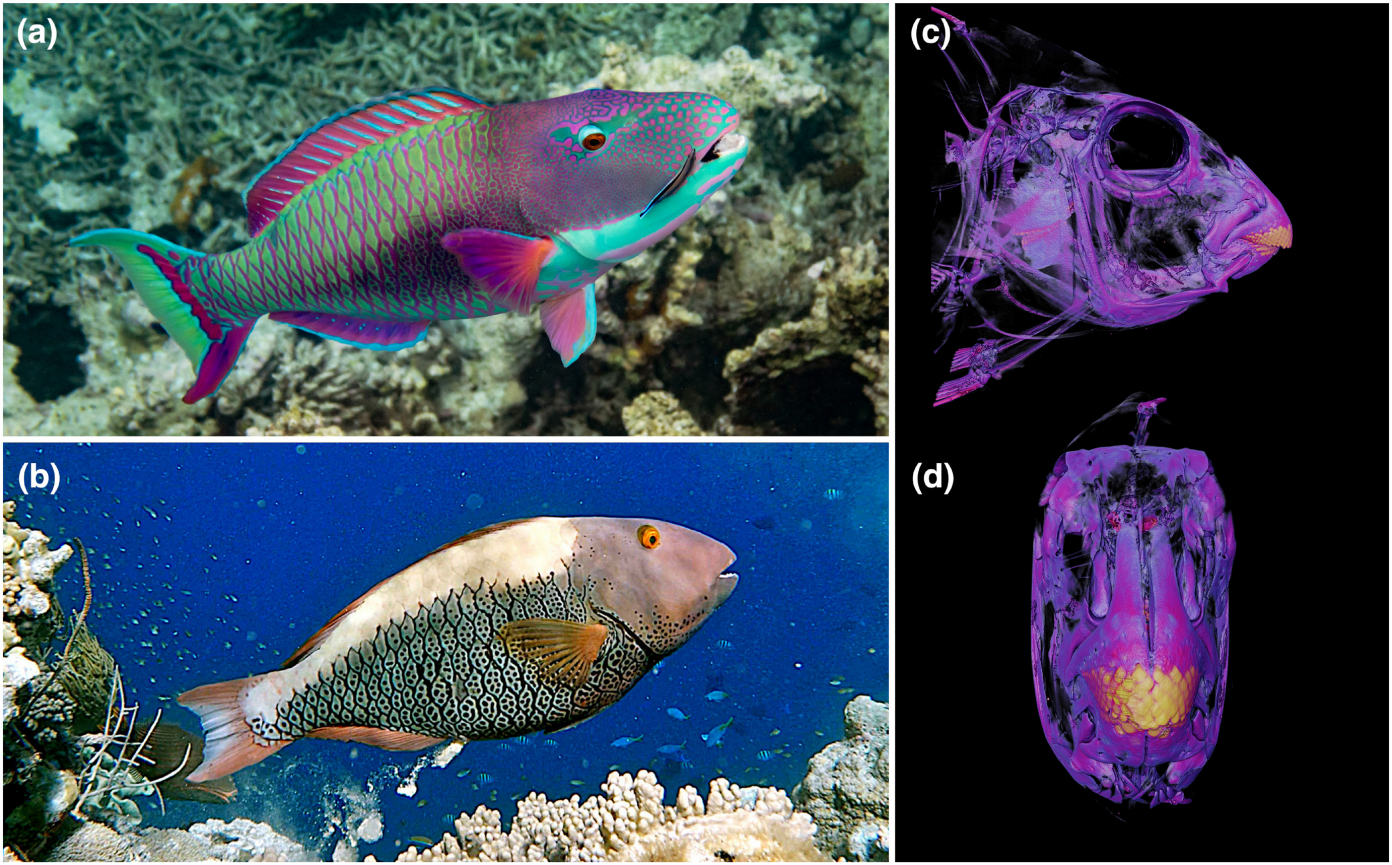
Spotted parrotfish (*Cetoscarus ocellatus*) demonstrating pronounced dimorphism in (a) male and (b) female body coloration. The beak‐like dentition typical of parrotfishes is highlighted in the micro‐CT scans (c, d). Specimen registration number FMNH 110797. Photographs provided by David Earles and (a), Adelma Hills, (b) and micro‐CT scans provided by Kory Evans (c, d).

The expansion of gene families putatively involved in pigmentation and sex determination is interesting in the context of the Scarini. Unlike most other labrid groups, protogyny and sexual dimorphism are not universal in parrotfishes, accounting for only ~70% of all species (Streelman et al., 2002; Taylor et al., 2018). The spotted parrotfish is among those that display prominent sexual dimorphism, diandry, and protogyny (Streelman et al., 2002) (Figure 1), yet the role of sexual selection in the spotted parrotfish, and parrotfishes more generally, is not fully understood. Previous studies have suggested that the parrotfishes represent a classic example of adaptive radiation, with habitat partitioning into coral reef and seagrass habitats being the primary driver for initial diversification, followed by more recent diversification of lineages as a result of natural and sexual selection (Streelman et al., 2002; Streelman & Danley, 2003). This model has been proposed particularly for reef‐inhabiting lineages exhibiting socio‐sexual behaviour, extreme dichromatism and other patterns relating to sexual selection as a primary driver of secondary diversification following niche partitioning (Alfaro et al., 2009; Kazancioglu et al., 2009; Streelman & Danley, 2003). In contrast, more recent studies suggest that diversification of the parrotfishes was not a result of a single dominant sequential process, but rather a consequence of both neutral (drift following allopatry of sister species) and adaptive (natural and sexual selection in sympatric species) forces (Choat et al., 2012).

**FIGURE 2:**
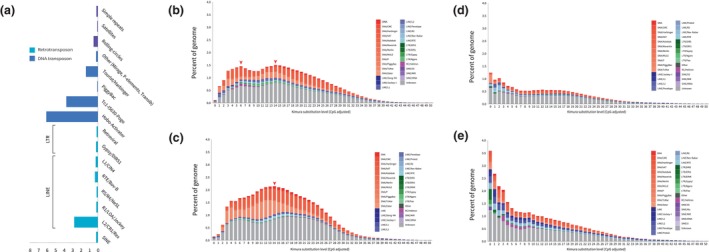
Transposable element (TE) evolution across four labrid genomes. (a) TE composition in the spotted parrotfish. TE expansion histories in (b) spotted parrotfish, (c) humphead wrasse, (d) corkwing wrasse and (e) ballan wrasse. *X* and *Y* axes indicate CpG‐adjusted Kimura substitution levels and percentage of TEs, respectively. Red arrows indicate independent waves of TE expansion in the genomes of the spotted parrotfish and humphead wrasse.

**FIGURE 3:**
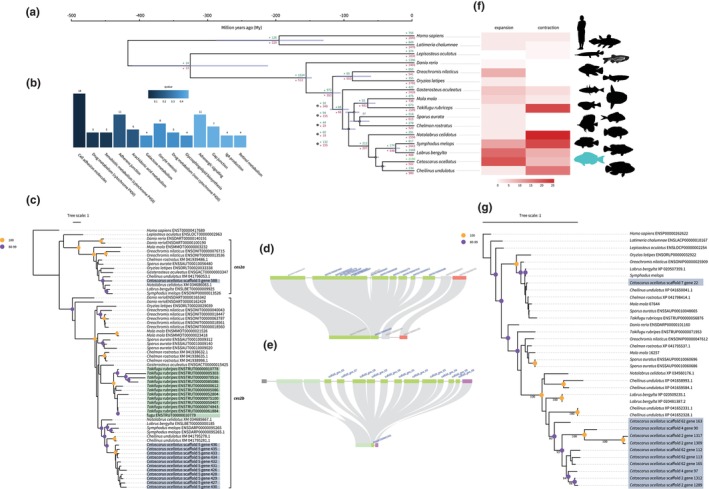
(a) Time‐calibrated phylogeny and gene families for various chordates. The coelacanth (Sarcopterygii), gar (non‐teleostean Actinopterygii) and human (Mammalia) were used as outgroup taxa. Unless specifically stated, all nodes had support values of 100%. Gene family expansion and contraction for each species and the most recent common ancestor are represented by arrows and values in green (for expansion) and red (for contraction), respectively. (b) Enrichment analysis of KEGG pathway for 572 expanded gene families in the spotted parrotfish. *X* axis indicates the pathway name, *Y* axis indicates the number of gene families enriched in each pathway. The colour bar indicates the *q*‐value. (c) Maximum‐likelihood phylogeny of *ces2* gene family in 16 species of comparative chordates. (d) Local synteny plot of *ces2b* from fugu (above) and stickleback (below). (e) Local synteny plot of *ces2b* from spotted parrotfish (above) and stickleback (below). (f) Heatmap of expanded or contracted gene families related to pigmentation genes, the numbers indicate the expanded or contracted gene family numbers in the respective species of the phylogeny. The coloured bar indicates the range of the gene family number. (g) Maximum‐likelihood phylogeny of *chst8* gene family. Bootstrap values (1000 bootstrap replicates) are reported as percentages. The orange circle and purple circle indicate bootstrap support of 100% and 80%–99%, respectively.

**FIGURE 4:**
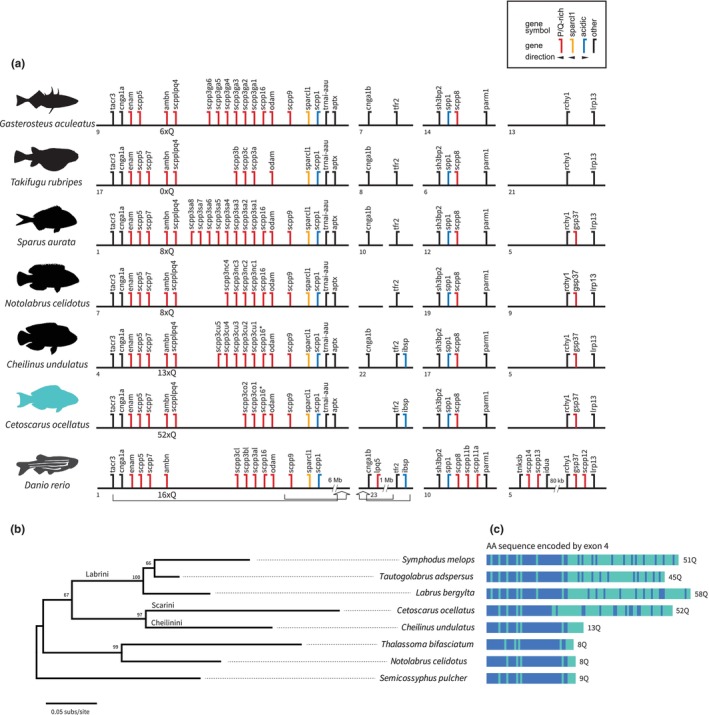
(a) Genomic arrangement of *scpp* genes in seven teleost fishes. Flags in different colours represent the order and transcriptional directions of *scpp* genes encoding proline/glutamine (P/Q)‐rich proteins (red), sparcl1 (yellow), acidic proteins (blue) (Mikami et al., 2022) and other genes (black), as summarized in the legend. Numbered horizontal lines represent chromosomes. In zebrafish (*Danio rerio*), the original *scpp* gene cluster on chromosome 1 and chromosome 23 was split by an inversion (arrow) and separated by a 6 Mb sequence and a 1 Mb sequence, respectively. In the zebrafish genome, *idua* and *rchy1* are separated by an 80 kb sequence. (b) Maximum‐likelihood gene tree of *ambn* and (c) the total number of glutamine residues (Q) encoded by exon 4 for the labrid species compared. Bootstrap values are given at nodes. Amino acid sequences are schematically shown here (see Supplemental Information for full amino acid sequences) with Gln residues in green and non‐Gln amino acids in blue. Values at nodes denote bootstrap support (1000 bootstrap replicates).

The spotted parrotfish genome exhibits expansions in a high proportion of PRG families (~25%) compared with the genomes of other species. We detected four copies of *pax3*, an evolutionarily conserved member of the paired‐box transcription factor family (Barr et al., 1999). This gene is mainly expressed in muscle cells, as well as in the skin, eyes, caudal fin, and scales, and plays an important role in the differentiation, migration and proliferation of melanin and xanthophores (Minchin & Hughes, 2008; Roberts et al., 2009). We also identified expansions of *chst8* in the spotted parrotfish genome, which in many organisms is involved in the production of sex hormones and is responsible for the sulfation and modification of luteinizing hormones (Cabral et al., 2012; Dumitrescu & Collins, 2008).

In other groups of coral reef fishes, selection acting on pigmentation genes has been cited as evidence of sexual selection. For example, in the widely sympatric Caribbean hamlet genus *Hypoplectrus*, strongly selected regions in the genome corresponding to pigmentation (*sox10*), patterning (*hoxc13a*), photoreceptor development (*casz1*), and visual sensitivity (*sws* and *lws* opsins) were thought to facilitate speciation in syntopic species despite extremely low divergence elsewhere in the genome (Hench et al., 2019). Expansion of opsin *sws2b*, *lws1,* and *rh2* genes in at least one other labrid species (Liu et al., 2021) suggests that visual acuity and colour perception are key drivers of evolution in labrids, likely conferring an evolutionary advantage on these fishes with complex social biology that rely strongly on colouration patterns for correct identification of conspecifics (Kazancioglu et al., 2009).

Additionally, we detected positive selection in *pak7*, *igf1,* and *rock1*, genes that were putatively identified as being involved in sex differentiation or sex determination signalling pathways (Gegenhuber et al., 2022; Reinecke, 2010). Positive selection was also detected in genes putatively involved in gonadal development (*col9a2*) (Piprek et al., 2018) and social behaviours (*pak7*) (Strochlic et al., 2012). In other vertebrates, *igf1* has been shown to accelerate the development of testicular tissue (Rahaie et al., 2018), whereas *rock1* has been shown to be tightly involved in oestrogen signalling (Huang et al., 2020). In labrids, sex change and sequential hermaphroditism are tightly regulated by social hierarchy, with sex change suppressed in the presence of a dominant terminal‐phase male. Because individuals frequently live in highly competitive and hierarchical social groups, it is critical that individuals rapidly assert and maintain behavioural dominance in order to achieve gonadal and morphological changes that lead to higher reproductive success (Lamm et al., 2015). In addition to morphological change in gonadal tissue, sex change is accompanied by the development of secondary male sexual characteristics, such as male‐specific colour patterns and external morphological traits. In some species, the initial dominance established by the highest‐ranking female following the removal of a terminal male to development of fully functional testes can happen in as little as 10 days (Lamm et al., 2015; Warner & Swearer, 1991).

While the selection and expansion of several gene families related to pigmentation and sex differentiation are consistent with sexual selection being an evolutionary driver, more work is needed to link functionality and traits, particularly since many of these genes exhibit pleiotropy across the lifespan of an animal. Comparative genomics with other scarine labrids that do not display apparent protogyny (e.g. *Leptoscarus*) or prominent sexual dimorphism (e.g. several species of *Scarus*) would provide better insight into the potential evolutionary roles of pigmentation and sex differentiation in scarine evolution.

### 4.3 A putative role of poly‐glutamine residues in teeth biomineralization

The beak‐like teeth of parrotfishes consist of two upper dental plates, and two lower ones, each comprising ~15 rows of teeth that are fused together. The biting ends of these teeth are biomineralized with a fluorapatite enameloid with a remarkable hardness of up to 7.3 GigaPascals, making this one of the stiffest and hardest biominerals on earth (Marcus et al., 2017). Analyses of known teeth‐forming genes such as *scpp3* could elucidate the evolution of this key phenotype.

Expression of *scpp3* genes is highly diverse across fishes. In pufferfishes for example, expression of *scpp3a* and *scpp3b* is concentrated in the oral epithelial cells covering the jaw and the pharyngeal epithelial cells overlying dental epithelium (Kawasaki et al., 2005). In the spotted gar, *scpp3cl* and *scpp3dl* are significantly expressed in the jaws but not in the skin, despite having ganoid scales covered with enamel (ganoin) (Mikami et al., 2022). Sticklebacks (family Gasterosteidae), while lacking scales entirely, possess six copies of *scpp3* genes (Figure 4a). In contrast, *scpp3* is absent in seahorses, which lack scales and teeth (Lin et al., 2016; Zhang et al., 2020). These studies suggest that *scpp3* is involved in tooth formation, but not in scale formation, although the reason for variations in the number of *scpp3* genes remains to be determined.

While the repertoire of *scpp* genes and their copy numbers showed minimal variation across comparative labrid species, analysis of amino acid sequences encoded by *scpp* genes revealed large differences in one particular gene, *ambn*. In mammals, *ambn* encodes a dental enamel matrix protein. Similarly, *ambn* is expressed during dental and scale enamel formation in non‐teleostean fishes such as gars and bichirs (Kawasaki et al., 2021, 2022). Although enamel was secondarily lost from teeth and scales in teleosts, *ambn* is still expressed in dental epithelial cells during teeth formation (Kawasaki et al., 2021), suggesting that *ambn* is somewhat involved in teeth formation of modern teleosts. The *ambn* gene in the spotted parrotfish encodes for 363 amino acids, while the *ambn* orthologue in its closest phylogenetic relative, the humphead wrasse (Figure 4b), encodes 309 amino acids. This difference is largely due to the number of glutamine (Gln) residues encoded by exon 4 (Figure 4c). In the spotted parrotfish, exon 4 of *ambn* encodes for 52 Gln residues, 12 of which are uninterrupted. In contrast, the *ambn* orthologue in the humphead wrasse encodes only 12 Gln residues. In other teleosts, exon 4 of *ambn* encodes 6–8 Gln residues in the gilthead seabream and stickleback. Curiously, the exon is missing entirely in the pufferfish genome (Figure 4a).

Comparison of exon 4 of *ambn* across labrid species reveals that it encodes between 45 and 58 Gln residues in three Labrini species, namely the ballan wrasse, corkwing wrasse, and the cunner. In contrast, this exon encodes only 8–9 Gln residues in two other species, blue‐head wrasse and sheepshead wrasse (Figure 4c). Among non‐labrid teleosts, exon 4 of *ambn* encodes 16 (zebrafish) or fewer Gln residues. To our knowledge, a large exon 4 of *ambn* encoding for 26 or more Gln residues has not been reported previously. These findings are the first for the following labrids: spotted parrotfish (tribe Scarini: with 52 Gln residues), ballan wrasse, corkwing wrasse, and the cunner (*T. adspersus*) (tribe Labrini: with 45–58 Gln residues). Phylogenetic reconstruction suggests a sister relationship between the Scarini and the Cheilinini, with the two sharing a sister relationship with the Labrini (Hughes et al., 2023) (Figure 4b). Given that poly‐Gln residues were not detected in the humphead wrasse, it appears that a rare poly‐Gln expansion encoded by exon 4 of *ambn* occurred once in the common ancestor of the Scarini + Cheilinini + Labrini, but was secondarily lost in the Cheilinini.

In vertebrates, mineralization and skeletal growth are accomplished through proteins containing polyproline repeat elements (Jin et al., 2009). In particular, glutamine plays an important role in biomineralization and in the formation of enamel and hydroxyapatite (Jin et al., 2009). Compaction of these polyproline motifs is especially important during enamel and apatite formation. Within these motifs, glutamine has been shown to greatly influence the compaction and function of these polyproline helices. Experimental replacement of even as few as 5 Gln residues with substitutes (e.g. alanine) has been shown to not only dramatically alter the diameter and density of these helices but also reverse the effect of macromolecular compaction. These results indicate that Gln residues play a pivotal role in compaction of polyproline helices as they occur in many biological systems including biominerals (Jin et al., 2009).

We hypothesize that the poly‐Gln expansion encoded by exon 4 of *ambn* in the parrotfish facilitates compaction of polyproline motifs expressed in the upper dentine layers of the parrotfish's beak. However, why this poly‐Gln expansion is retained in the Labrini is unclear. Members of this tribe typically feed on hard‐shelled crustaceans and molluscs, and having highly biomineralized teeth may help facilitate the breakdown of shelled prey (Ouannes‐Ghorbel & Bouain, 2006). However, the absence of poly‐Gln expansion in other labrid groups that feed on similar prey items is curious (e.g. the sheepshead wrasse, tribe Hypsigenyini), suggesting multiple, independent evolutionary pathways for biomineralization of teeth within the Labridae.

## 5 CONCLUSIONS

The evolution of parrotfishes has traditionally been considered adaptive radiation diverging primarily along axes of habitat colonization, followed by trophic morphology, and reproduction as a result of sexual selection (Streelman & Danley, 2003). This dominant theory has eroded in some respects, with more focused studies finding evidence for interactions among evolutionary mechanisms such as ecological and sexual selection (Choat et al., 2012). Our study provides genomic insight into some of these hypotheses. A recent genomic study of the whitespot parrotfish (*Scarus forsteni*; Liang et al., 2024) has obtained results that are consistent with the findings of our study, but further genome sequencing of this group will provide a platform for an improved understanding of parrotfish evolution.

For instance, the expansion and selection of detoxification genes in the spotted parrotfish provide new insight into a possible role in the metabolism of harmful dietary compounds. While the exact dietary elements thought to be responsible for the increased presence of these detoxification genes are yet unknown, our results provide some support for earlier trophic studies showing that the dietary repertoire of the spotted parrotfish includes corals, sponges and cyanobacterial filaments. At least some of these taxa are known to harbour secondary metabolites that deter grazing in coral reef fishes (Nicholson & Clements, 2023a, 2023b).

We also find selection and expansion of PRG families and genes involved in sex differentiation typical of protogynous species. While these results provide some evidence of sexual selection, our study focused on a well‐known protogynous species displaying strong dimorphism between sexes. Comparisons with additional taxa that do not display obvious sexual dimorphism and diandry are needed to corroborate these findings.

Although genome‐scale data can provide a powerful source of information for the study of evolution, they have limitations when used at the exclusion of other data types. For example, we still do not fully understand the role of poly‐Gln expansion in the *ambn* gene of the spotted parrotfish. While its role here is putatively linked to teeth biomineralization, investigating the functionality of this expansion is a priority for future research.

## AUTHOR CONTRIBUTIONS

**Yi‐Kai Tea:** Conceptualization (equal); formal analysis (equal); funding acquisition (equal); writing – original draft (equal); writing – review and editing (equal). **Yulu Zhou:** Formal analysis (equal); investigation (equal); writing – original draft (equal); writing – review and editing (equal). **Kyle M. Ewart:** Conceptualization (equal); data curation (equal); formal analysis (equal); investigation (equal); writing – original draft (equal); writing – review and editing (equal). **Guo Cheng:** Formal analysis (equal); methodology (equal); writing – review and editing (equal). **Kazuhiko Kawasaki:** Data curation (equal); formal analysis (equal); writing – original draft (equal); writing – review and editing (equal). **Joseph D. DiBattista:** Writing – original draft (equal); writing – review and editing (equal). **Simon Y. W. Ho:** Conceptualization (equal); funding acquisition (equal); investigation (equal); supervision (equal); writing – original draft (equal); writing – review and editing (equal). **Nathan Lo:** Conceptualization (equal); funding acquisition (equal); supervision (equal); writing – original draft (equal); writing – review and editing (equal). **Shaohua Fan:** Conceptualization (equal); funding acquisition (equal); investigation (equal); supervision (equal); writing – original draft (equal); writing – review and editing (equal).

## FUNDING INFORMATION

The study was funded by a USyd–Fudan Partnership Collaboration Award to Y. K. T., J. D. D., S. Y. W. H., N. L. and S. H. F. Y. K. T. is supported by the Chadwick Biodiversity Fellowship. S.H.F. is supported by grants from the National Key R&D Program of China (2020YFE0201600 and 2021YFC2500202), the National Natural Science Foundation of China (32370686), the 111 Project (B13016) and Shanghai Municipal Science and Technology (2017SHZDZX01 and 19410741100).

### OPEN RESEARCH BADGES

This article has earned an Open Data badge for making publicly available the digitally‐shareable data necessary to reproduce the reported results. The data is available at [https://doi.org/10.5061/dryad.j6q573nkz].

## Supporting information


Appendix S1.

## Data Availability

The *Cetoscarus ocellatus* genome assembly and annotation is available from the Dryad Digital Repository: https://doi.org/10/5061/dryad.j6q573nkz. The genome assembly is also archived on GenBank under accession JBAMN000000000 (BioSample SAMN40150312), an all associated sequence data are archived on the SRA database under the same BioProject (PRJNA1081164).
