# Supplementary material for: The spotted parrotfish genome provides insights into the evolution of a coral reef dietary specialist (Teleostei: Labridae: Scarini: Cetoscarus ocellatus)
Source: Ecol Evol. 2024 Mar 12;14(3):e11148. doi: 10.1002/ece3.11148 (PMC10932694; doi:10.1002/ece3.11148)
Supplement: Supplementary file 1 — Appendix S1. [file ECE3-14-e11148-s001.pdf]

Supplemental Information for:

The spotted parrotfish genome provides evolutionary insight into the ecological adaptation of a keystone dietary specialist

Yi-Kai **Tea**, Yulu **Zhou**, Kyle M. **Ewart**, Guo **Cheng**, Kazuhiko **Kawasaki**, Joseph D. **DiBattista**, Simon Y. W. **Ho**, Nathan **Lo** & Shaohua **Fan**

TABLE OF CONTENTS:

**Supplemental Information**	**Page 1**
**Supplemental Table S1**	**Page 2**
**Supplemental Table S2**	**Page 5**
**Supplemental Table S3**	**Page 5**
**Supplemental Table S4**	**Page 6**
**Supplemental Table S5**	**Page 6**
**Supplemental Figure S1**	**Page 7**
**Supplemental Figure S2**	**Page 7**
**Supplemental Figure S3**	**Page 8**
**Supplemental Figure S4**	**Page 8**
**Supplemental Figure S5**	**Page 9**
**Supplemental Figure S6**	**Page 9**

Supplemental Table S1. ARKS/LINKS parameter sweep. The combination of parameters that resulted in the least number of scaffolds (*n* = 1616) was chosen for further scaffolding with Hi-C data.

c	m	z	l	a	e	# Scaffolds
5	50-10000	500	5	0.3	30000	2670
3	50-10000	500	5	0.3	30000	2213
2	50-10000	500	5	0.3	30000	2213
3	50-10000	500	5	0.9	30000	2209
3	50-10000	500	3	0.9	30000	2209
3	50-10000	500	3	0.9	30000	1894
3	50-10000	500	2	0.9	30000	1894
3	50-10000	1000	3	0.9	30000	1894
3	50-10000	500	3	0.9	50000	1731
3	50-10000	500	3	0.9	70000	1696
3	50-10000	500	3	0.9	90000	1711
3	10-250	500	3	0.9	70000	1616

Supplemental Table S2. Sequencing data statistics for *C. ocellatus* genome assembly.

Reads used	Method	# Contigs	N50 (bp)	Assembly length (bp)	Largest contig (bp)
PacBio	Canu	12,055	856,743	1,739,239,040	8,519,865
PacBio	purge_dups	2,671	1,190,296	1,366,641,025	8,519,865
PacBio (polishing)	Arrow	2,671	1,190,369	1,367,488,635	8,522,749
TELL-seq	ARCS/LINKS	1,215	4,977,857	1,367,634,235	22,842,595
Hi-C	SALSA2	727	29,377,150	1,367,634,235	22,842,595
PacBio (polishing)	Arrow	727	29,376,842	1,367,892,171	57,876,293
TELL-seq (polishing)	FreeBayes (1x)	727	29,375,828	1,367,827,497	57,874,231
TELL-seq (polishing)	FreeBayes (2x)	727	29,375,761	1,367,823,589	57,874,017

Supplemental Table S3. Benchmarking Universal Single-Copy Orthologs (BUSCO) evaluated for the completeness of the *Cetoscarus. ocellatus* genome.

BUSCO	Gene number	Percent
Complete BUSCOs	3583	98.5%
Complete and single-copy BUSCOs	3533	97.1%
Complete and duplicated BUSCOs	50	1.4%
Fragmented BUSCOs	23	0.6%
Missing BUSCOs	34	0.9%
Total BUSCO groups searched	3640	-

Supplemental Table S4. Repetitive element annotations in the *Cetoscarus ocellatus* genome.

Type of repeat	No. of repeats	Length (bp)	% of genome
RNA transposon	327,496	91,931,682	6.72
LTR	89,745	22,452,492	1.64
LINE	203,447	64,647,816	4.73
SINE	34,304	4,831,374	0.35
DNA transposon	926,502	244,174,663	17.85
hAT	432,555	102,831,648	7.52
Tc1/Mariner	164,223	53,879,591	3.94
PiggyBac	8,327	2,214,314	0.16
Tourist/Harbinger	110,129	29,403,491	2.15
Other	29,974	6,011,885	0.44
Rolling-circles	17,716	4,336,491	0.32
Unclassified	1,310,145	289,722,042	21.18
Small RNA	35,170	6,017,454	0.44
Simple repeats	329,220	15,920,597	1.16
Low complexity	44,689	2,423,921	0.18
Total interspersed repeats	2,564,143	625,828,387	45.75

Supplemental Table S5. Positively selected genes detected by branch-site model.

Gene ID	Gene name	P-value	Corrected P-value
ENSDARG00000032849	ndrg1a	0.001104733	0.040766809
ENSDARG00000099280	utp20	0.01151901	0.164233163
ENSDARG00000007278	ect2	0.003145383	0.077475199
ENSDARG00000040046	snai2	0.000690838	0.028995422
ENSDARG00000099461	asb10	3.52E-10	3.31E-07
ENSDARG00000007526	ndufs2	0.005536594	0.111894167
ENSDARG00000063310	oxr1b	0.006243653	0.117540054
ENSDARG00000008852	elp4	0.000693302	0.028995422
ENSDARG00000011615	mybpc3	0.00655803	0.118675124
ENSDARG00000019008	erp44	0.001716879	0.054742493
ENSDARG00000074691	mms22l	1.19E-05	0.00223492
ENSDARG00000025269	pdcd6ip	0.001987568	0.05937465
ENSDARG00000099972	nup153	0.000885879	0.034733824
ENSDARG00000012403	ercc6l2	0.001832591	0.055627994
ENSDARG00000044134	iars2	0.004464348	0.096573604
ENSDARG00000075824	virma	0.003844628	0.085124584
ENSDARG00000099298	xrcc5	0.000519995	0.025093114
ENSDARG00000069013	prdx4	0.011880786	0.166862976
ENSDARG00000051816	aass	0.007678477	0.130187069
ENSDARG00000039456	acbd6	7.91E-06	0.001722773
ENSDARG00000099651	bin3	0.007453044	0.128684659
ENSDARG00000059387	fgf7	0.000475421	0.025093114
ENSDARG00000068280	GRB14	0.00025392	0.015929257
ENSDARG00000079854	scyl1	0.004716919	0.099744286
ENSDARG00000055076	nxf1	0.00695381	0.12282416
ENSDARG00000101199	rbp4	0.000224354	0.015608905
ENSDARG00000059177	tax1bp3	7.92E-05	0.007450404
ENSDARG00000007657	ccnh	0.013046194	0.179415599
ENSDARG00000102615	fra10ac1	0.002537151	0.06540983
ENSDARG00000039914	gapdhs	0.003068472	0.076998181
ENSDARG00000012896	anos1a	0.006983095	0.12282416
ENSDARG00000036820	mgll	8.24E-06	0.001722773
ENSDARG00000090108	si:ch1073-174d20.1	1.93E-05	0.00290074
ENSDARG00000057064	enpep	0.009305226	0.144730878
ENSDARG00000104314	nrg1	0.000626178	0.028058759
ENSDARG00000075215	vezt	5.10E-06	0.001370005
ENSDARG00000033852	mad1l1	7.70E-05	0.007450404
ENSDARG00000077092	elk4	0.001745244	0.054742493
ENSDARG00000104295	arhgap21a	0.00027504	0.016697569
ENSDARG00000052515	calcoco2	0.000911708	0.035017023
ENSDARG00000103099	EARS2	0.009248002	0.144730878
ENSDARG00000094132	igf1	2.80E-09	1.75E-06
ENSDARG00000013721	g6pca.2	0.008457586	0.136044241
ENSDARG00000009733	kif21b	0.006829595	0.122412356
ENSDARG00000058801	rev3l	1.62E-06	0.000608915

ENSDARG00000069044	agpat4	0.002214523	0.061617389
ENSDARG00000017676	mmp2	0.003755898	0.085124584
ENSDARG00000020984	slc16a10	3.40E-05	0.003766756
ENSDARG00000079055	si:dkey-85a20.4	0.011257937	0.163727614
ENSDARG00000104103	rps6kc1	2.45E-05	0.003065836
ENSDARG00000018923	fat2	0.000165774	0.012999419
ENSDARG00000004055	uhrf1bp1l	0.000241447	0.015669101
ENSDARG00000076781	trim45	0.005867259	0.114682049
ENSDARG00000063661	nuak2	0.009207367	0.144730878
ENSDARG00000101959	etv1	0.000544784	0.025632096
ENSDARG00000092609	aup1	2.61E-05	0.003065836
ENSDARG00000056932	tfip11	0.001286131	0.045669793
ENSDARG00000102458	hip1rb	0.002260632	0.061617389
ENSDARG00000069673	chid1	0.003017885	0.076752168
ENSDARG00000037433	fmr1	0.00560794	0.111894167
ENSDARG00000013667	tbck	0.000501644	0.025093114
ENSDARG00000079166	ace	0.009697497	0.147182973
ENSDARG00000063299	pcloa	0.002421171	0.063286711
ENSDARG00000075707	nid2a	0.009435437	0.145553219
ENSDARG00000079241	wdr90	0.001738516	0.054742493
ENSDARG00000057100	zwilch	0.000177384	0.013353498
ENSDARG00000036637	arl11	0.006307941	0.117540054
ENSDARG00000021239	apaf1	0.000512937	0.025093114
ENSDARG00000016010	nup107	0.008358614	0.136044241
ENSDARG00000013072	mmp15b	0.003575272	0.084108283
ENSDARG00000101220	polrmt	0.000420916	0.023298938
ENSDARG00000103868	e2f1	0.002160565	0.061617389
ENSDARG00000059263	hoxd12a	2.16E-05	0.00290074
ENSDARG00000032199	gpc3	0.005648218	0.111894167
ENSDARG00000077928	ccdc173	0.013246206	0.180647537
ENSDARG00000012066	dcn	0.014170957	0.19049815
ENSDARG00000086158	ccbe1	0.003232204	0.077987274
ENSDARG00000052893	rx3	0.011396555	0.163727614
ENSDARG00000001857	aff4	0.00135758	0.047314172
ENSDARG00000043079	MMP23B	0.015011891	0.197569086
ENSDARG00000101631	etfa	0.01218812	0.16991142
ENSDARG00000040644	pxylp1	0.014592158	0.194769082
ENSDARG00000013333	ndufa10	0.007604831	0.13011174
ENSDARG00000102478	ADAMTS7	0.000685606	0.028995422
ENSDARG00000000568	ell	0.001519192	0.051055713
ENSDARG00000054746	uggt1	0.000834356	0.033409732
ENSDARG00000075299	prxl2c	0.00627251	0.117540054
ENSDARG00000043301	gtf3c6	0.004026603	0.088117054
ENSDARG00000074129	edem3	0.003750875	0.085124584
ENSDARG00000074836	setmar	0.00049985	0.025093114
ENSDARG00000087633	si:dkey-11o18.5	0.008258091	0.136044241
ENSDARG00000070677	fezf2	5.23E-05	0.005463652
ENSDARG00000009001	pdia6	0.000992011	0.037339287
ENSDARG00000074033	adamtsl2	0.005288629	0.109375823
ENSDARG00000002084	lamb2	9.33E-32	1.76E-28

ENSDARG00000058993	rock1	0.009278318	0.144730878
ENSDARG00000075159	meltf	0.01306054	0.179415599
ENSDARG00000017321	kdr	0.005947201	0.114682049
ENSDARG00000026024	mospd2	0.00227032	0.061617389
ENSDARG00000098764	musk	0.000232226	0.015608905
ENSDARG00000079377	arhgef38	8.49E-05	0.007605922
ENSDARG00000086362	pou6f2	0.000136079	0.011134845
ENSDARG00000101831	irx1a	0.001613832	0.053284759
ENSDARG00000007744	tsr1	4.02E-06	0.001262216
ENSDARG00000040072	scpep1	0.011709302	0.165691032
ENSDARG00000024492	col9a2	2.00E-05	0.00290074
ENSDARG00000029729	clybl	0.000720966	0.029496894
ENSDARG00000038456	mrps35	0.000597525	0.027427853
ENSDARG00000109443	EML4	0.003169814	0.077475199
ENSDARG00000005453	foxp2	0.000347466	0.01981612
ENSDARG00000090447	mtbp	0.00545707	0.111632666
ENSDARG00000042637	tert	0.011270819	0.163727614
ENSDARG00000059610	gpr146	0.011377107	0.163727614
ENSDARG00000045131	id4	0.005182306	0.10836778
ENSDARG00000059923	slc25a47a	0.001135072	0.04108087
ENSDARG00000062531	mapk8ip3	0.014766535	0.195708587
ENSDARG00000102877	bphl	0.005971754	0.114682049
ENSDARG00000100965	ep400	0.013847387	0.187487637
ENSDARG00000019438	rnf13	4.41E-08	2.08E-05
ENSDARG00000103977	terf2ip	0.001794745	0.05537229
ENSDARG00000030154	pak7	0.002210825	0.061617389
ENSDARG00000056193	nme7	2.15E-05	0.00290074
ENSDARG00000062765	nsd3	0.002058719	0.060539219
ENSDARG00000078801	msantd1	0.002324567	0.061617389
ENSDARG00000102394	ap1g1	0.004658733	0.099633365
ENSDARG00000059798	kcnq1.1	0.010647554	0.157785015
ENSDARG00000068288	lamc2	0.000335551	0.01973459
ENSDARG00000059792	trpm5	0.002323296	0.061617389
ENSDARG00000052344	tbl3	0.00711557	0.123995391
ENSDARG00000011723	C2H5orf22	0.009617574	0.147156709
ENSDARG00000035515	tm2d2	0.006421343	0.118435279
ENSDARG00000100549	CABZ01079427.1	0.003808316	0.085124584
ENSDARG00000060633	usp16	0.010085432	0.150641133
ENSDARG00000114022	WDR1	0.007870767	0.131086584
ENSDARG00000053026	kif19	0.000210236	0.015217856
ENSDARG00000073985	pctp	0.001447473	0.049529896
ENSDARG00000079363	lrig3	0.008420166	0.136044241
ENSDARG00000063161	ppwd1	0.007747583	0.130187069
ENSDARG00000071823	adarb2	0.006481846	0.118435279
ENSDARG00000069133	mstnb	0.0001276	0.010915601
ENSDARG00000052343	spata18	0.003482746	0.082968707
ENSDARG00000023026	pkp2	0.003743275	0.085124584
ENSDARG00000060515	zdhhc9	0.009906093	0.149146136

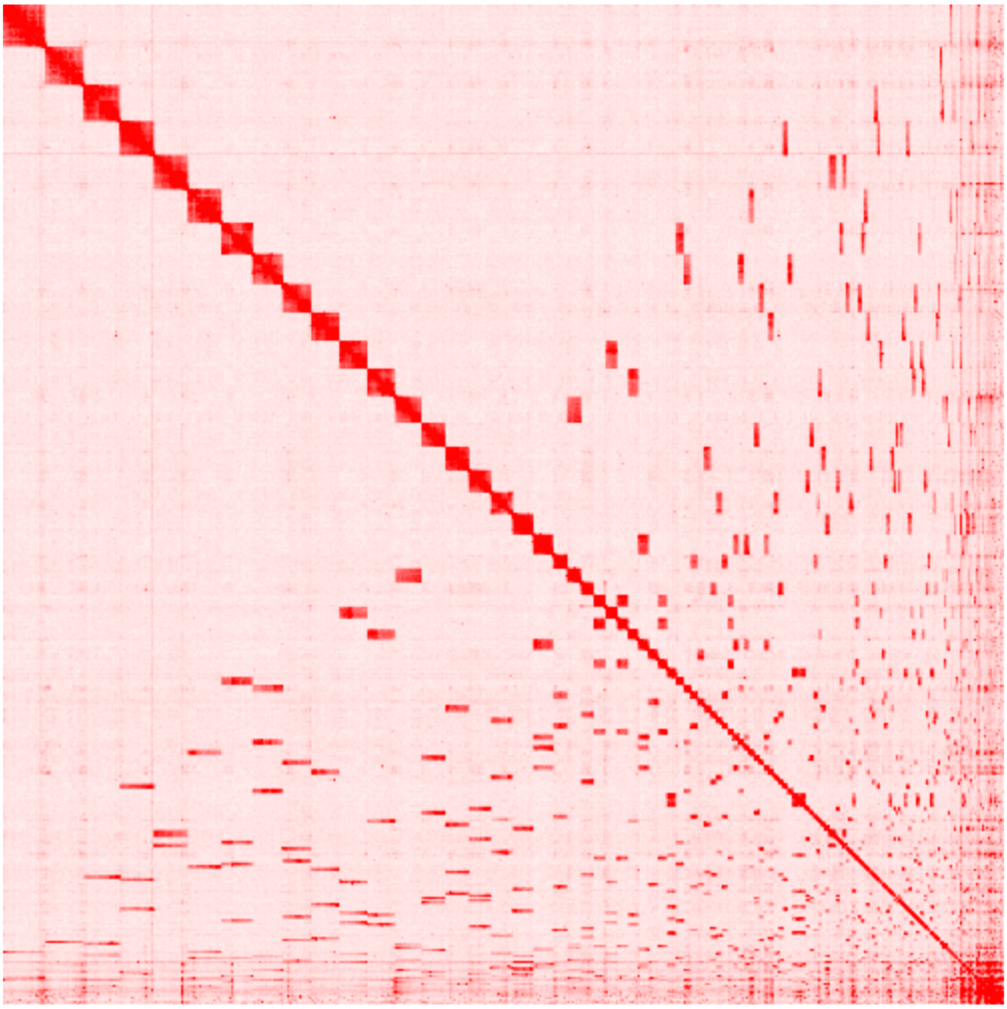

Supplemental Figure S1. Hi-C contact map generated by Juicebox. Darker shades indicate more frequent contacts between loci.

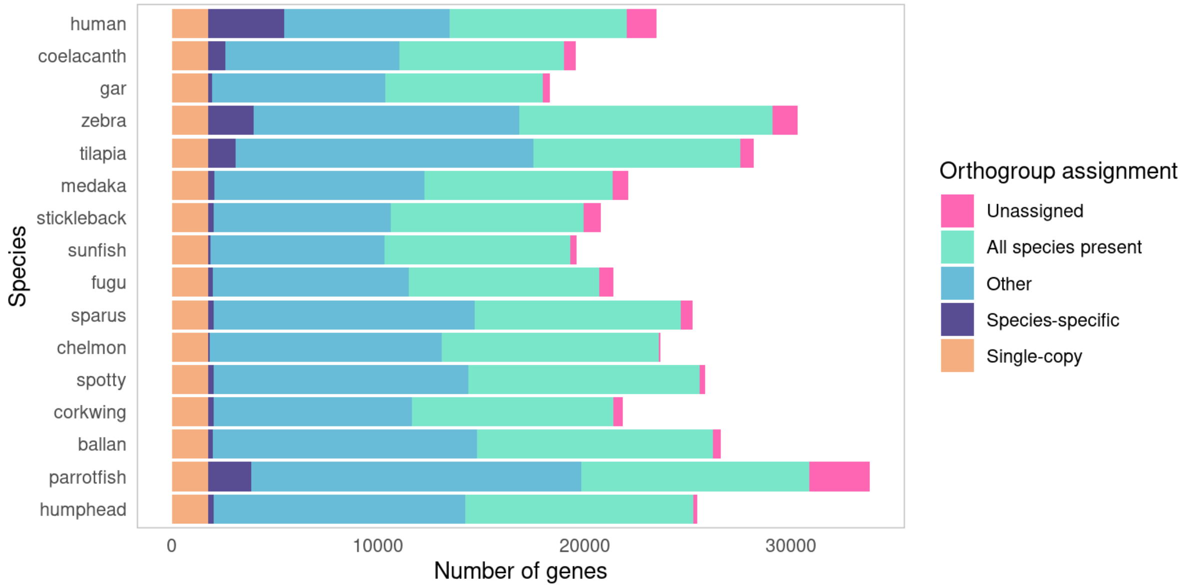

Supplementary Figure S2. OrthoFinder assignment of annotated proteins. Orthogroups assigned as "Other" consist of groups where not all sequences are present, or contain a variable number of (paralogous) genes.

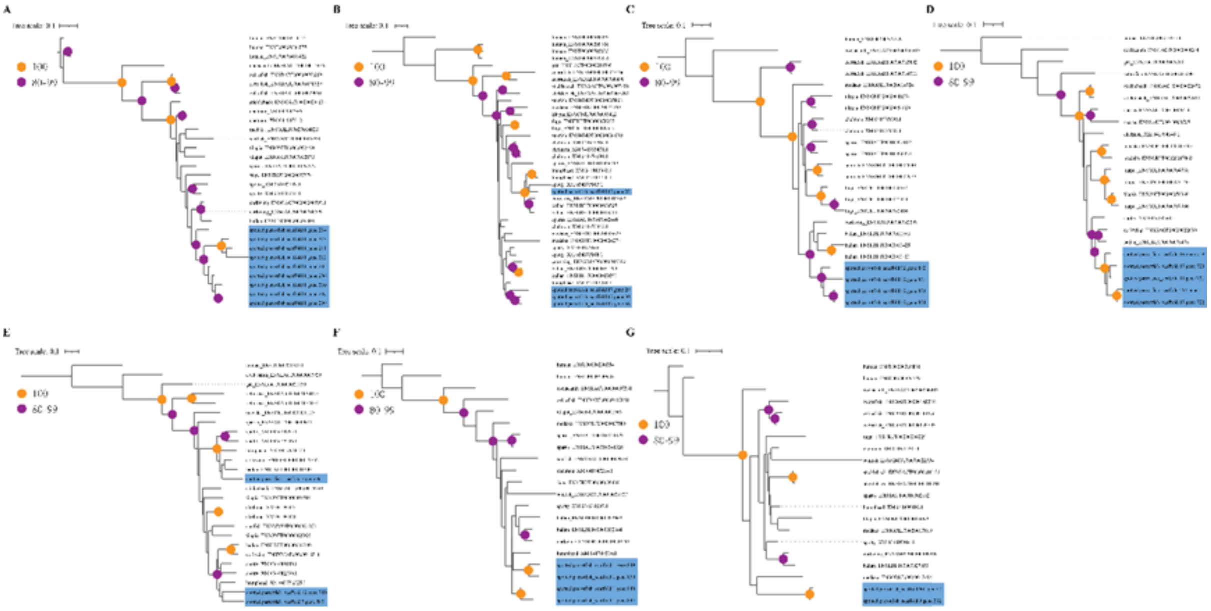

Supplemental Figure S3. Maximum-likelihood phylogeny of significantly expanded xenobiotics metabolism related gene families in spotted parrotfish. (A): Maximum-likelihood phylogeny of *gsta* gene families. (B): Maximum-likelihood phylogeny of *adh1* gene families. (C): Maximum-likelihood phylogeny of *fmo5* gene families. (D): Maximum-likelihood phylogeny of *cyp3a65* gene families. (E): Maximum-likelihood phylogeny of *nat10l* gene families. (F): Maximum-likelihood phylogeny of *cbr1* gene families. (G): Maximum-likelihood phylogeny of *gstm* gene families. Genes in spotted parrotfish are highlighted in blue. The orange circle and purple circle indicate bootstrap support 100 and bootstrap support from 80 to 99 respectively. Bootstrap values (1000 bootstrap replicates) are reported as percentages.

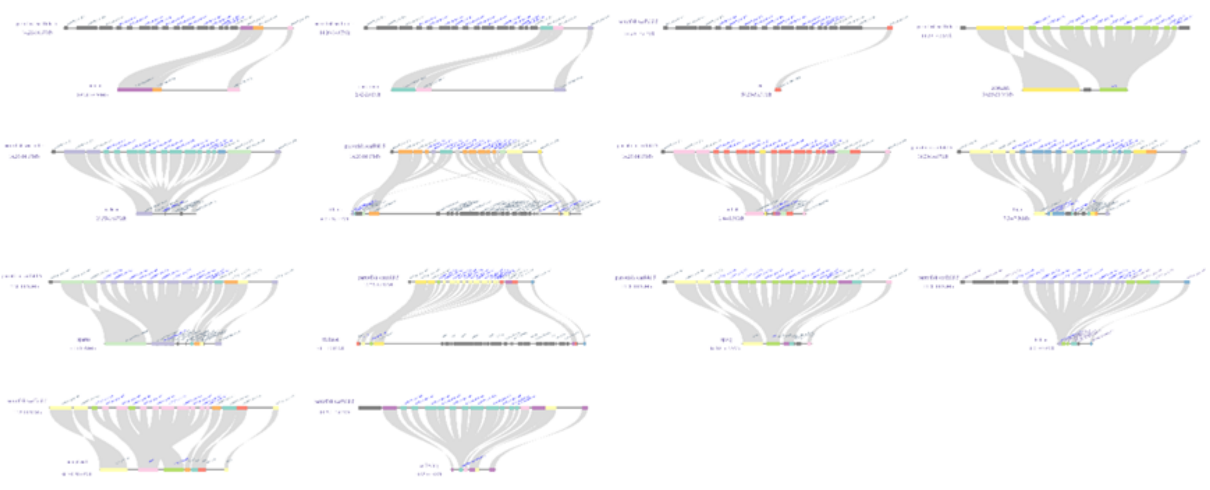

Supplemental Figure S4. Local synteny plot of *ces2b* from spotted parrotfish and other comparative species. *ces2b* genes are highlighted in blue.

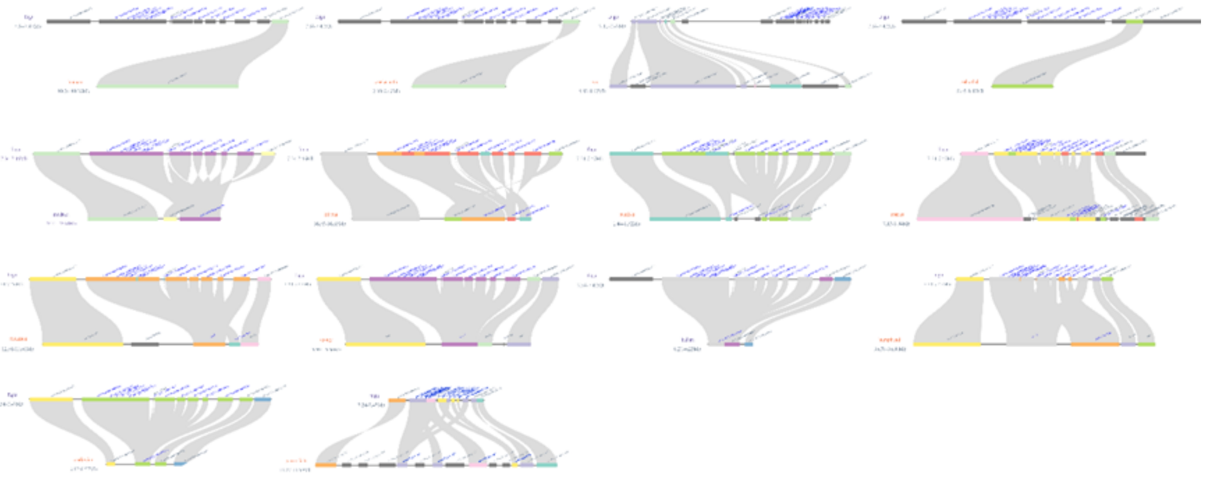

Supplemental Figure S5. Local synteny plot of *ces2b* from Japanese pufferfish and other fourteen species. *ces2b* genes are highlighted in blue.

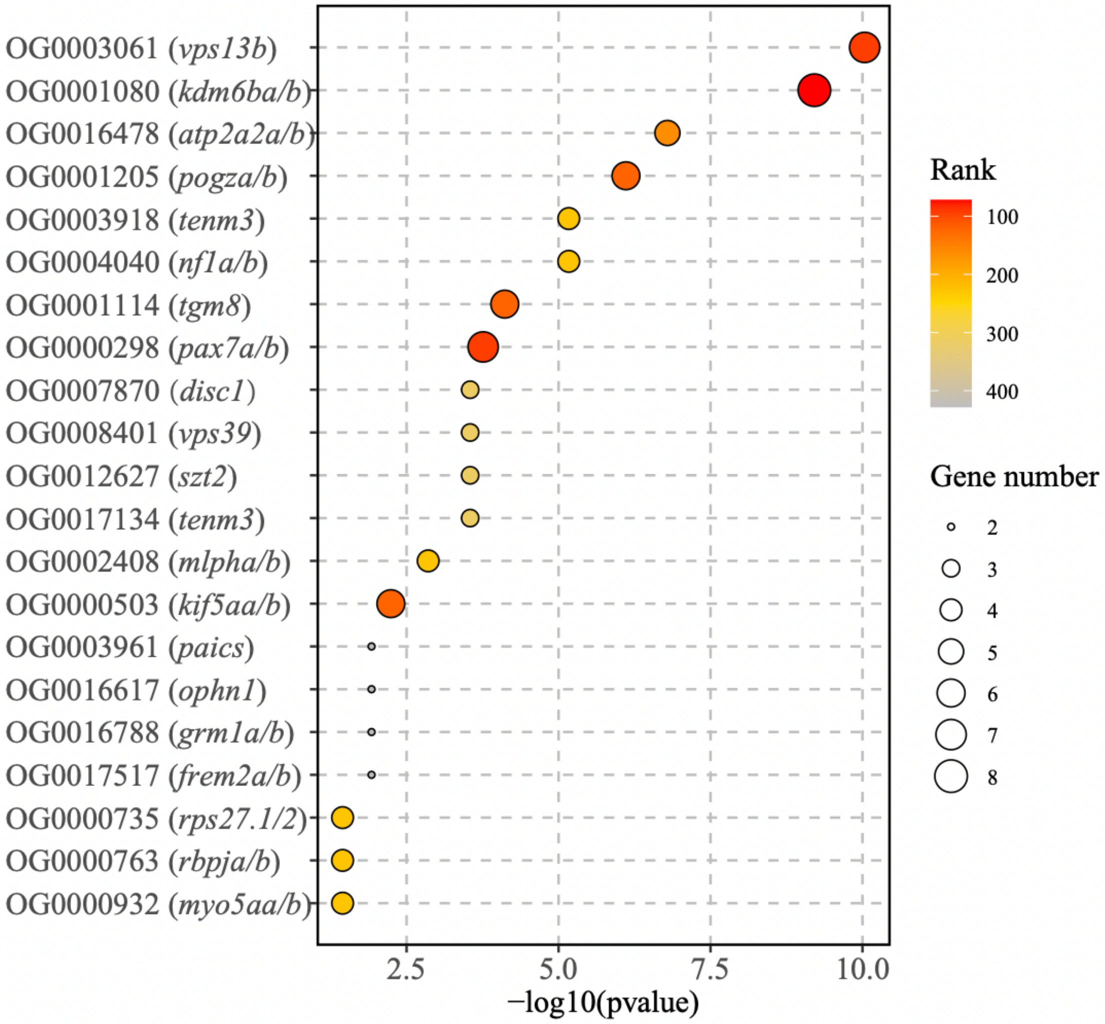

Supplemental Figure S6. Plot of significantly expanded pigmentation-related genes in the spotted parrotfish genome. P-values calculated by cafe analysis are represented on the x-axis. The size of each dot represents the number of genes belonging to each gene family. A colour gradient is used to rank all expanded gene families. Gene family IDs are labelled on the y-axis, with their annotated gene symbols in parentheses.

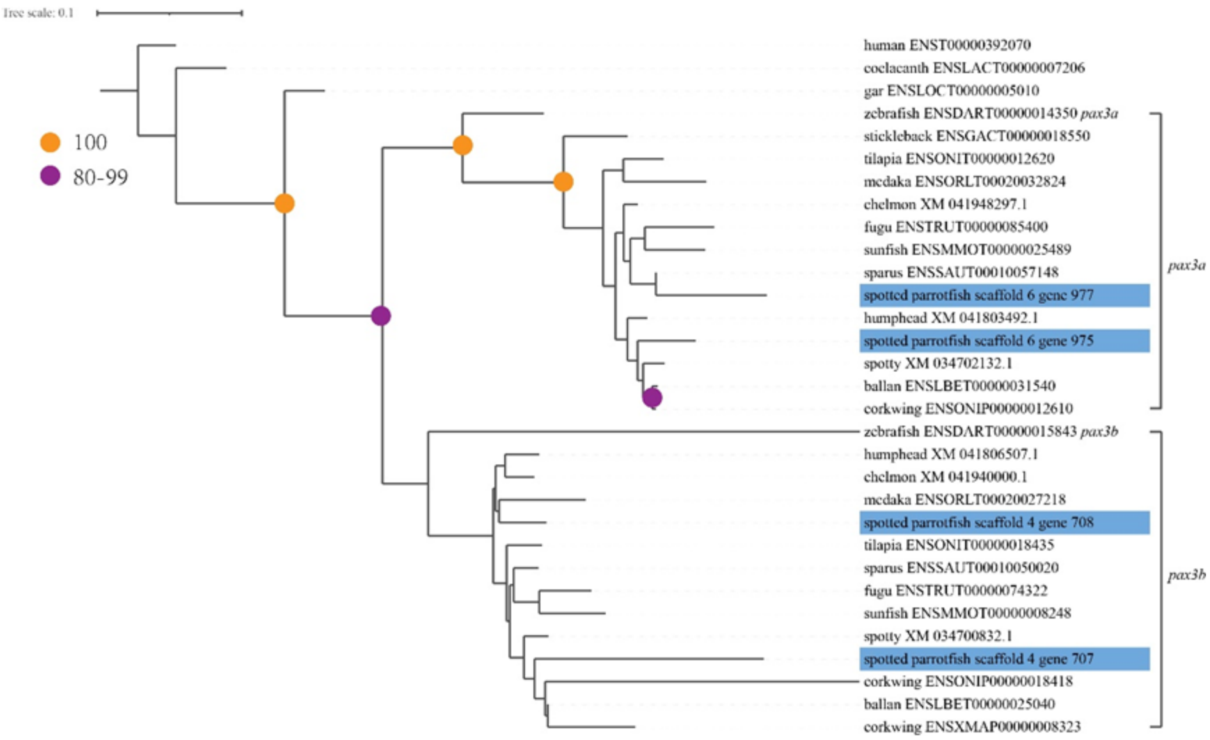

Supplemental Figure S7. Maximum-likelihood phylogeny of significantly expanded pigmentation related gene family *pax3* in spotted parrotfish. Genes in spotted parrotfish are highlighted in blue. The orange circle and purple circle indicate bootstrap support 100 and bootstrap support from 80 to 99 respectively. Bootstrap values (1000 bootstrap replicates) are reported as percentages.
